# *MLgrating*: a program for simulating multilayer gratings for tender X-ray applications

**DOI:** 10.1107/S1600577524006271

**Published:** 2024-08-01

**Authors:** Andrew Walters, Shengyou Wen, Qiushi Huang, Zhanshan Wang, Hongchang Wang, Kawal Sawhney

**Affiliations:** a Diamond Light Source Ltd Harwell Science and Innovation Campus Didcot OxfordshireOX11 0DE United Kingdom; b Key Laboratory of Advanced Micro-Structured Materials MOE, Institute of Precision Optical Engineering, School of Physics Science and Engineering Tongji University Shanghai200092 People’s Republic of China; c Shanghai Professional Technical Service Platform for Full-Spectrum and High-Performance Optical Thin Film Devices and Applications Tongji University Shanghai200092 People’s Republic of China; d Shanghai Frontiers Science Center of Digital Optics Tongji University Shanghai200092 People’s Republic of China; Deutsches Elektronen-Synchrotron, Germany

**Keywords:** multilayer gratings, grating efficiency simulations, blazed gratings, laminar gratings

## Abstract

Multilayer gratings are increasingly popular optical elements at X-ray beamlines, as they can provide much higher photon flux in the tender X-ray range compared with traditional single-layer coated gratings. While there are several proprietary software tools that provide the functionality to simulate the efficiencies of such gratings, until now the X-ray community has lacked an open-source alternative. Here *MLgrating* is presented, a program for simulating the efficiencies of both multilayer gratings and single-layer coated gratings for X-ray applications. *MLgrating* is benchmarked by comparing its output with that of other software tools and plans are discussed for how the program could be extended in the future.

## Abstract 2

*MLgrating*, a MATLAB program for simulating multilayer grating efficiencies, is presented.

## 1. Introduction

It is an ongoing challenge for X-ray beamlines to provide both high flux and high resolving power (*E*/Δ*E*) in the tender X-ray range (1–5 keV). In recent years, several X-ray facilities have developed active research programmes concerning multilayer gratings, as their efficiencies are typically an order of magnitude larger than those of single-layer coated gratings in the tender X-ray range (Choueikani *et al.*, 2014 ▸; Voronov *et al.*, 2016 ▸; Sokolov *et al.*, 2019 ▸).

Double-crystal monochromators (DCMs) which use the Si(111) reflection can also be employed in the tender X-ray range – at least above 2 keV – but they suffer from several drawbacks compared with multilayer plane-grating monochromators (PGMs). Firstly, to satisfy the Bragg condition, the grazing angles need to be large (>20°), leading to high power densities which can deform the crystal, potentially impacting upon performance. Secondly, the large grazing angles mean that their transmission of p-polarized light is poor, especially around 2800 eV where the grazing angle is about 45°. Thirdly, multilayer PGMs have the great advantage of tunability: it is possible to provide high resolving powers by closing the corresponding exit slit, but if more moderate resolving powers are required then one can increase the exit slit opening to simultaneously lower the resolving power and increase the sample flux. With a DCM, the relationship between resolving power and flux is fixed for a given energy.

**Figure 1:**
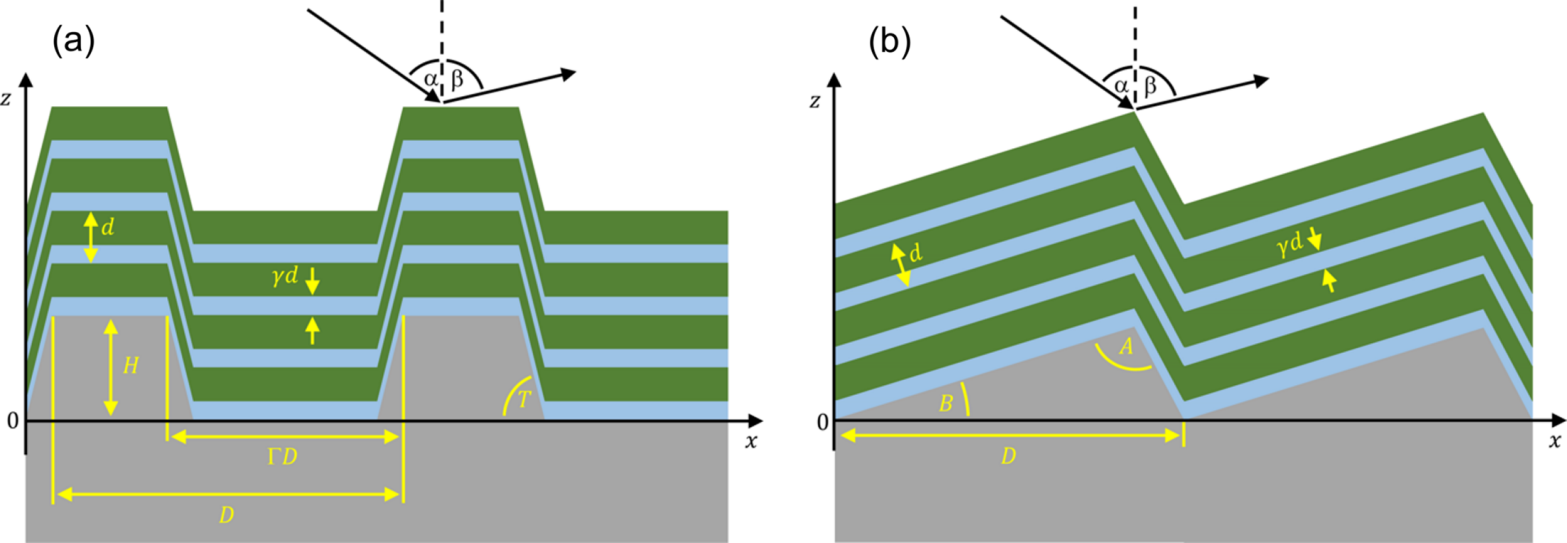
Schematic cross-sectional views of multilayer gratings with all relevant parameters labelled. A multilayer laminar grating is shown in (*a*), while a multilayer blazed grating is presented in (*b*).

Multilayer gratings fall into two primary categories: multilayer laminar gratings and multilayer blazed gratings. Schematic views of these gratings are presented in Fig. 1 ▸, with the labelled parameters described in Table 1 ▸. In all of the following we assume that the laminar or blazed profile is present in the underlying substrate (normally made of silicon for X-ray applications), and a multilayer is then deposited onto the grating structure. While the theoretical maximum efficiency of a multilayer blazed grating can be very close to the reflectivity of the corresponding multilayer with no underlying grating structure, the theoretical maximum efficiency of multilayer laminar gratings is intrinsically limited, typically being at most ∼80% of the reflectivity of the corresponding multilayer (Yang *et al.*, 2017 ▸). The current record grating efficiencies for multilayer gratings operating in first order at 3 keV is ∼60% for a multilayer blazed grating (Sokolov *et al.*, 2019 ▸) and ∼45% for a multilayer laminar grating (Wen *et al.*, 2024 ▸). A detailed and contemporary description of the development of multilayer gratings at synchrotron facilities can be found in a recent study (Wen *et al.*, 2024 ▸) and the references therein.

While single-layer coated gratings can provide high efficiencies at lower energies where the grazing angles are below the critical angle of total external reflectivity, multilayer-coated gratings can enhance the efficiency at higher energies via Bragg diffraction from a multilayer coating. This means that two diffraction conditions need to be met simultaneously for each wavelength λ: the grating equation 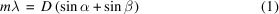
and the generalized Bragg equation for multilayer gratings
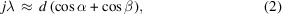
where α(β) is the grating incidence (diffraction) angle relative to the grating normal as presented in Fig. 1 ▸. Parameters *m*, *D* and *d* are defined in Table 1 ▸, while *j* is the Bragg diffraction order for the multilayer. We note that the approximation symbol in equation (2) is required because there are additional terms in the exact treatment which are related to the refractive indices of the materials as well as the details of the grating structure (Yang *et al.*, 2017 ▸). In most practical cases the additional terms which have been excluded from equation (2) are rather small and can be neglected.

In general, the grating parameters (see Table 1 ▸) affect both the resolving power and flux of an X-ray beamline. There are well established methods to model the resolving power of a PGM-based X-ray beamline, either analytically (Follath & Senf, 1997 ▸; Walters *et al.*, 2022 ▸) or through ray tracing (Sanchez del Rio *et al.*, 2011 ▸). However, simulating the grating efficiency, and therefore predicting the photon flux, is far more challenging computationally.

There are several commercially available software packages for simulating grating efficiencies. These include *UNIGIT* (Osires Optical Engineering, 2017 ▸), *PCGrate* (International Intellectual Group, 2020 ▸), *GSolver* (Grating Solver Development, 2023 ▸) and *RSoft DiffractMOD* (Synopsys, 2023 ▸). Alternatively there are freely available programs such as *REFLEC* (Schäfers & Krumrey, 1996 ▸), *GD-Calc* (Johnson, 2022 ▸), *RETICOLO* (Hugonin & Lalanne, 2005 ▸), *RawDog* (Kajtár, 2014 ▸), *RCWA* (Podolskiy, 2017 ▸) and *PPML* (Zanotto, 2022 ▸). One should be aware that the commercial solutions tend to provide superior performance, either by providing additional algorithms for calculating the grating efficiencies such as the C-method (Chandezon *et al.*, 1980 ▸) or in overall computational performance. However, most commercially available software packages have been designed for different scientific applications (typically in the UV–Vis wavelength range) which can make them inconvenient to use for X-rays, especially as one often needs to define the length scales describing the grating and the photon wavelength in micrometres when nanometres is a far more practical unit for X-ray applications.

At Diamond Light Source and several other X-ray facilities, *REFLEC* has been regularly used for many years thanks to its well designed and intuitive interface, as well as the ease of installation. However, one encounters difficulties when using *REFLEC* in simulating gratings which contain more than one material type, such as multilayer gratings. In addition, when designing new gratings for future beamlines, one would like to script grating efficiency simulations over a range of practical groove parameters such as the blaze angle *B* and the apex angle *A* for a blazed grating. Such scripting is extremely difficult to do in *REFLEC*.

Until now, no freeware has been developed that can straightforwardly simulate multilayer gratings for X-ray applications. Such a program would be of tremendous value to theX-ray community, especially in view of the increasing use of these optical elements. We have therefore developed *MLgrating* (*MultiLayer grating*), a program written in MATLAB (MathWorks Inc., 2021 ▸), which uses *GD-Calc* (Johnson, 2022 ▸) to simulate both single-layer and multilayer grating efficiencies using the Rigorous Coupled-Wave (RCW) method (Neviere & Popov, 1999 ▸). *MLgrating* primarily functions as a preprocessor to *GD-Calc*: it uses the input parameters defined by the user (described in Table 1 ▸) to produce a MATLAB structure which fully describes the (multilayer) grating in the format required for *GD-Calc*. The grating parameters in *MLgrating* have been defined in a format that closely follows the established definitions from *REFLEC*. *MLgrating* is freely available on GitHub https://github.com/DiamondLightSource/MLgrating, and requires a MATLAB licence to run.

## 2. Benchmarking of *MLgrating*

*GD-Calc* uses cuboid blocks to represent any arbitrary 1D (or 2D) grating profile (Johnson, 2022 ▸). Our initial development of *MLgrating* therefore focused on simulating laminar gratings made of a single material with trapezoidal angles *T* of 90° (see Fig. 1 ▸), as these can be simulated efficiently with one block of height *H* and width Γ*D* combined with a substrate and a superstrate. Grating efficiency software packages which use the RCW method typically require the user to define *m*_*max*, the maximum grating diffraction order *m* [*cf.* equation (1)] for which grating efficiencies are simulated. Increasing *m*_*max* typically improves the accuracy of the simulations at the expense of requiring additional computation time.

**Figure 2:**
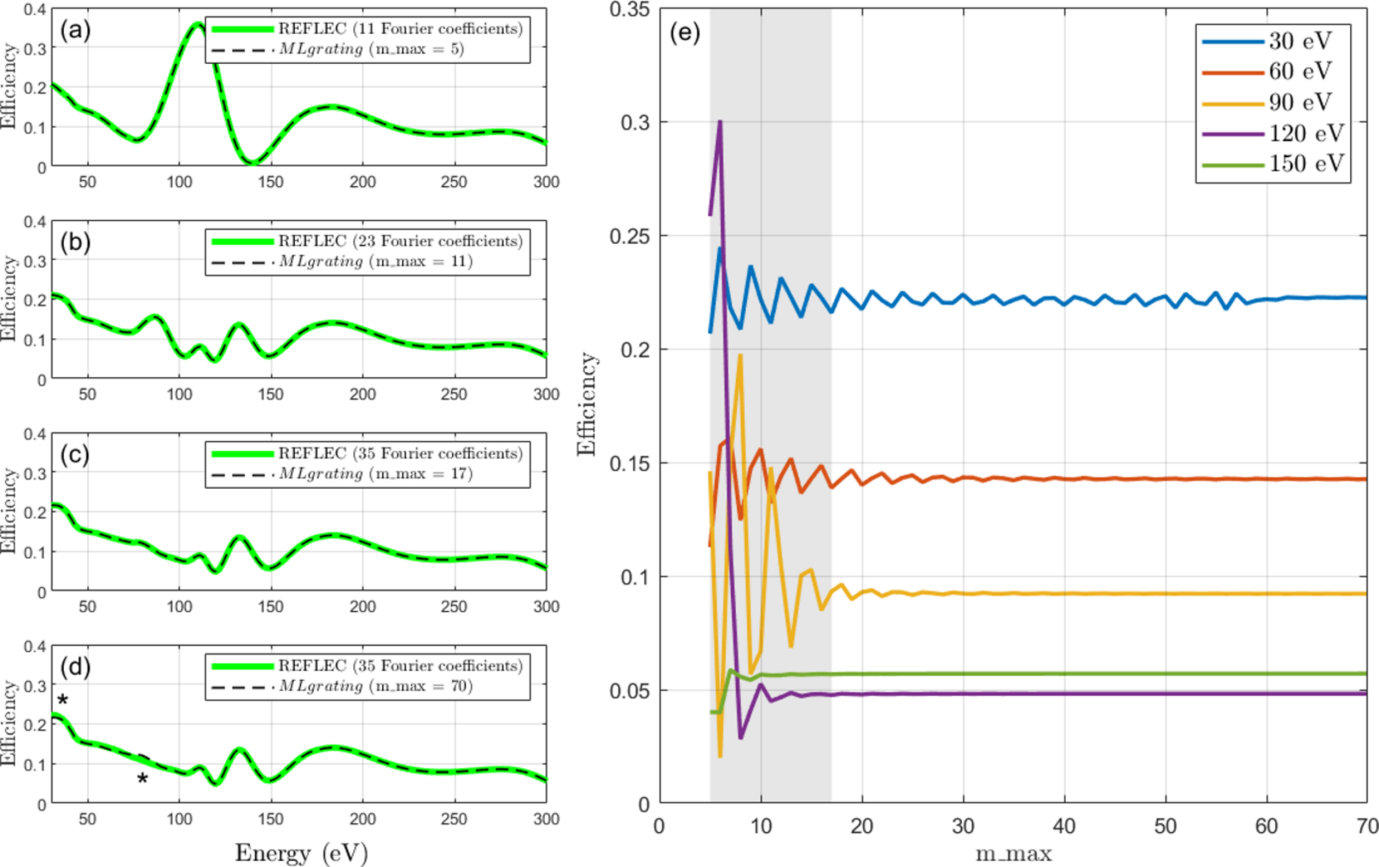
Convergence testing of *REFLEC* and *MLgrating* is presented as a function of *m*_*max*, the maximum diffraction order simulated. Here the grating was assumed to be made entirely of rhodium, *N* = 800 lines mm^−1^, *H* = 42.5 nm, Γ = 0.65, *T* = 90°, *m* = −1 and *c*_ff_ = 2. Panels (*a*)–(*c*) present grating efficiency simulations performed in *MLgrating* for different values of *m*_*max*: (*a*) 5, (*b*) 11 and (*c*) 17, together with corresponding simulations performed in *REFLEC*. In (*d*) a *MLgrating* simulation using *m*_*max* = 70 is presented, and compared with a *REFLEC* simulation using the maximum number of Fourier coefficients possible (equivalent to *m*_*max* = 17). The asterisks highlight regions where the two simulations significantly deviate from one another. In (*e*) the *MLgrating* grating efficiency versus *m*_*max* is plotted for various energies, showing how the grating efficiency simulation converges as *m*_*max* is increased.

Fig. 2 ▸ summarizes a detailed comparison of the convergence properties of *REFLEC* and *MLgrating* for an example laminar grating with *T* = 90°. The close agreement presented in Figs. 2 ▸(*a*)–2 ▸(*c*) between *REFLEC* and *MLgrating* provides strong evidence that the number of Fourier coefficients in *REFLEC* is equivalent to 2*m*_*max* + 1. This can be straightforwardly understood, as *m*_*max* as defined in *GD-Calc* defines a truncation limit to the diffraction orders, both positive and negative. This means that for a given *m*_*max*, the number of diffraction orders, or ‘Fourier coefficients’ in the parlance of *REFLEC*, that will be simulated will be 2*m*_*max* + 1 (including the zeroth order).

In Fig. 2 ▸(*d*) we compare a *REFLEC* simulation with the maximum number of Fourier coefficients allowed (35, equivalent to *m*_*max* = 17) with a simulation in *MLgrating* with *m*_*max* = 70. One can observe small differences between the two curves, showing that the simulation is not fully converged when *m*_*max* = 17. The convergence behaviour of this simulation is shown in more detail in Fig. 2 ▸(*e*). Here the grating efficiency at five example energies is presented as a function of *m*_*max*, clearly showing how the simulation converges with increasing *m*_*max*. The grey region in Fig. 2 ▸(*e*) is the region that is accessible within *REFLEC*. For this example, one needs to simulate with *m*_*max* > 60 to be well converged at the lowest energies, which is well beyond the capabilities of *REFLEC*. The number of diffraction orders that can be simulated in *GD-Calc* is limited only by the memory available to MATLAB.

Both *GD-Calc* and *REFLEC* are designed so that the refractive indices 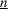 of the grating materials can be defined by the user by providing an input file. All simulations presented here have used the refractive indices available on the Center for X-ray Optics (CXRO) website maintained by Eric Gullikson at the Lawrence Berkeley National Laboratory (Henke *et al.*, 1993 ▸). *MLgrating* includes an input function which reads the refractive indices in the file format produced by the CXRO website. This function also performs the conversion from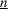 = 1 − δ − β to 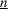 = *n* − *i*κ which is required for *GD-Calc*.

**Figure 3:**
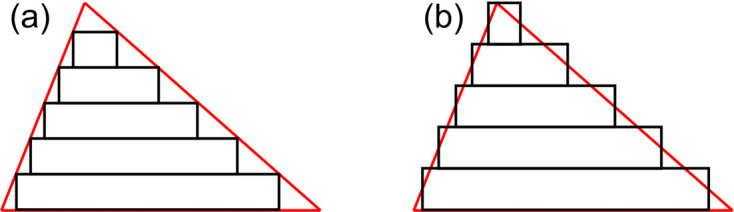
Comparison between different methods for approximating a blazed grating using the staircase approximation. (*a*) shows the approach presented in a recent work (Termini *et al.*, 2022 ▸), while (*b*) shows the approach implemented in *MLgrating*.

To simulate blazed gratings within the constraints of *GD-Calc* requires us to develop a staircase approximation to the blazed profile. A recent article concerning the design of UV blazed gratings for astronomy applications proposed a method for doing this in *GD-Calc* (Termini *et al.*, 2022 ▸). Their approach is presented in Fig. 3 ▸(*a*). However, the authors comment that this approach always leads to the total height of the blazed grating being smaller than its true height. Within this method they found that hundreds of slices were required for their grating efficiency simulations to be well converged. In *MLgrating* we take a significantly different approach, as presented in Fig. 3 ▸(*b*). Here we calculate the width of each slice so that its width matches the width of the exact blazed grating at the mid-point of the slice in height. The height of each slice is simply defined as *H*/*num*_*slices*, where *num*_*slices* is the number of slices defining the sloping interface. This means that the total height of the grating is always identical to the exact blazed grating, irrespective of the value of *num*_*slices*.

**Figure 4:**
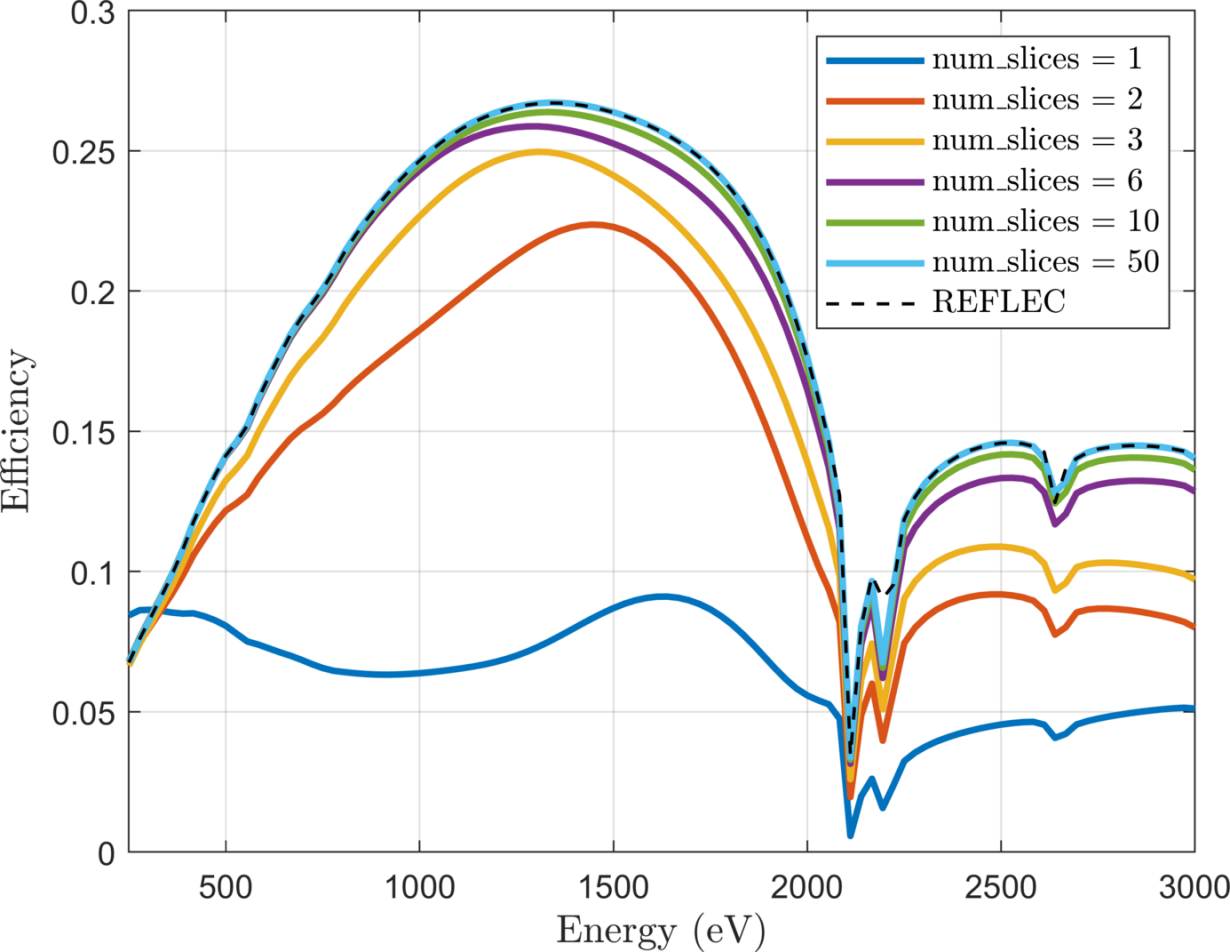
Convergence testing of *MLgrating* is presented as a function of the number of slices used to describe a sloping interface (*num*_*slices*). The details of the grating structure are described in the main text. The equivalent simulation performed in *REFLEC* is also shown.

In Fig. 4 ▸ we present convergence testing of *MLgrating* as a function of *num*_*slices* for an example blazed grating design. The grating simulated was assumed to be made entirely of platinum, with *N* = 600 lines mm^−1^, *B* = 0.49°, *A* = 175.62°,*m* = −1 and *c*_ff_ = 2.25. The maximum diffraction order *m*_*max* was set to 17. For this case we find that with *num*_*slices* = 6 the relative simulation error compared with *REFLEC* is already less than 10%. With *num*_*slices* = 10, this reduces to below 4%. Rather like the behaviour discussed earlier for *m*_*max*, when we increase the value of *num*_*slices* the simulation accuracy will increase, but the time taken for the simulation will also increase. We have therefore compared the speed of *MLgrating* with that of *REFLEC* on a typical modern laptop, and the results are shown in Table 2 ▸. We find that the simulations take a comparable amount of time if *num*_*slices* = 20. The relative error of the *MLgrating* efficiencies is <0.2% compared with *REFLEC* when *num*_*slices* = 20, so for this blazed grating *MLgrating* provides comparable performance with *REFLEC* in terms of both accuracy and speed. We note here that it is recommended that *MLgrating* users perform convergence testing firstly for *m*_*max* and then for *num*_*slices*. It is possible that there will be correlations between the two parameters, depending on the grating structure, so one should proceed with caution.

**Figure 5:**
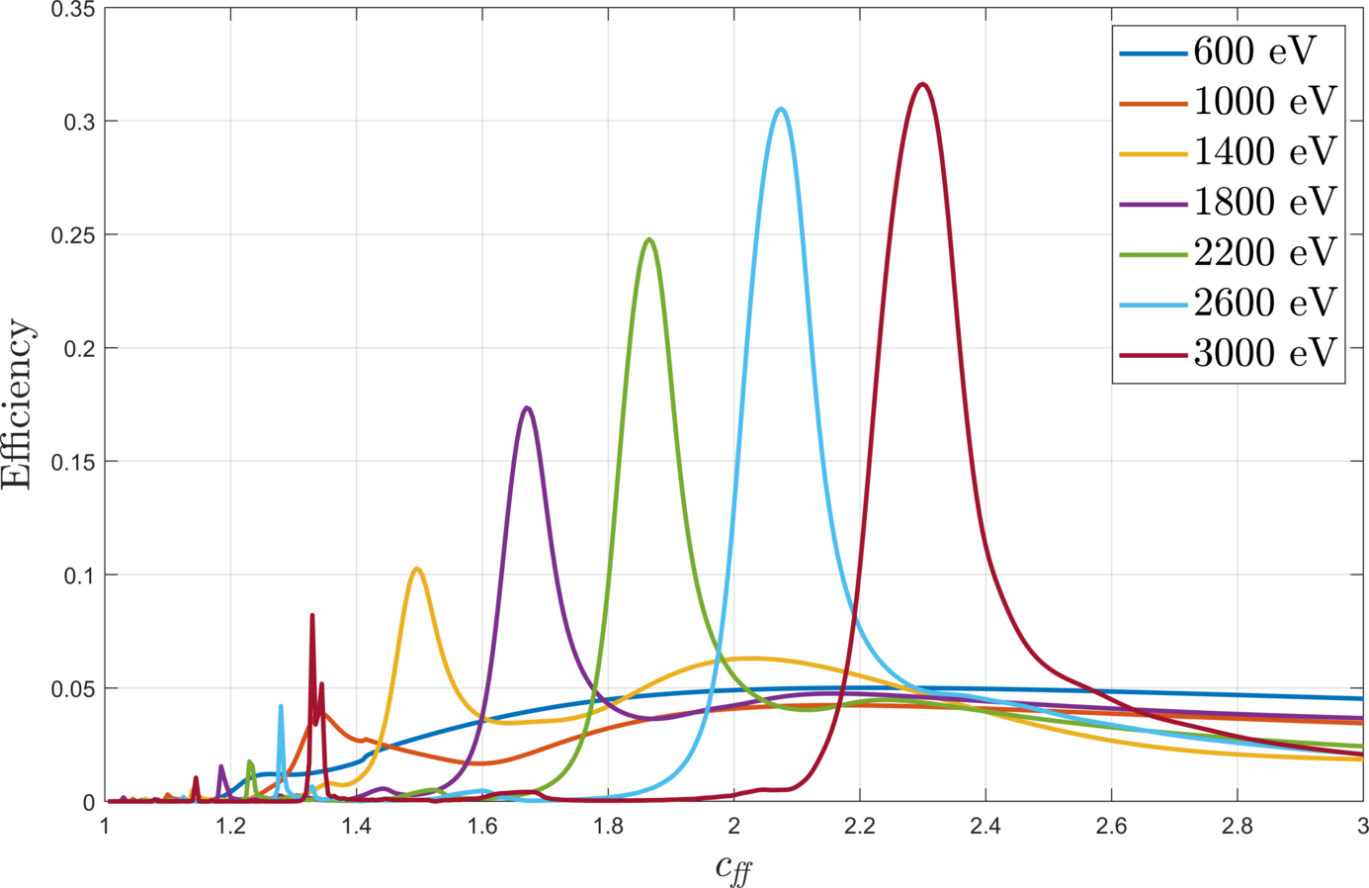
The efficiency of a multilayer laminar grating is plotted as a function of *c*_ff_ at several energies between 600 and 3000 eV inclusive. A Cr/C multilayer on a silicon grating was simulated [see the corresponding schematic in Fig. 1 ▸(*a*)], with *N* = 1200 lines mm^−1^, *H* = 7.5 nm, Γ = 0.74, *m* = −1, *d* = 10.5 nm, γ = 0.45 and *num*_*periods* = 50. The value of *m*_*max* was 3.

We now move on to testing of *MLgrating* in simulating multilayer gratings. We firstly simulated multilayer laminar gratings, as these are more trivial to simulate within the block structure used in *GD-Calc*. An illustrative set of example simulations is presented in Fig. 5 ▸. Here the grating efficiency at representative energies has been plotted as a function of the grating *c*_ff_. The multilayer coating presented here has been optimized to maximize the efficiency as if it was deposited on an existing uncoated laminar grating originally purchased for the B07 beamline at Diamond Light Source (Held *et al.*, 2020 ▸). For a collimated PGM beamline such as B07, *c*_ff_ can be varied over a wide range to tune the energy resolution, flux or vertical divergence depending on the experimental requirements. One can take advantage of that flexibility to design a multilayer grating which can provide high efficiencies within that *c*_ff_ range. Fig. 5 ▸ shows how the multilayer coating on the grating means that the multilayer grating can only be operated at a particular *c*_ff_ if one wants to maximize the flux. As described earlier, with a multilayer grating we need to satisfy both equations (1) and (2) simultaneously, which can only be done at a particular *c*_ff_ for a given photon energy. One can also observe from Fig. 5 ▸ that the optimal value of *c*_ff_ is evolving as a function of energy. This is a natural consequence of Bragg’s law [equation (2)].

**Figure 6:**
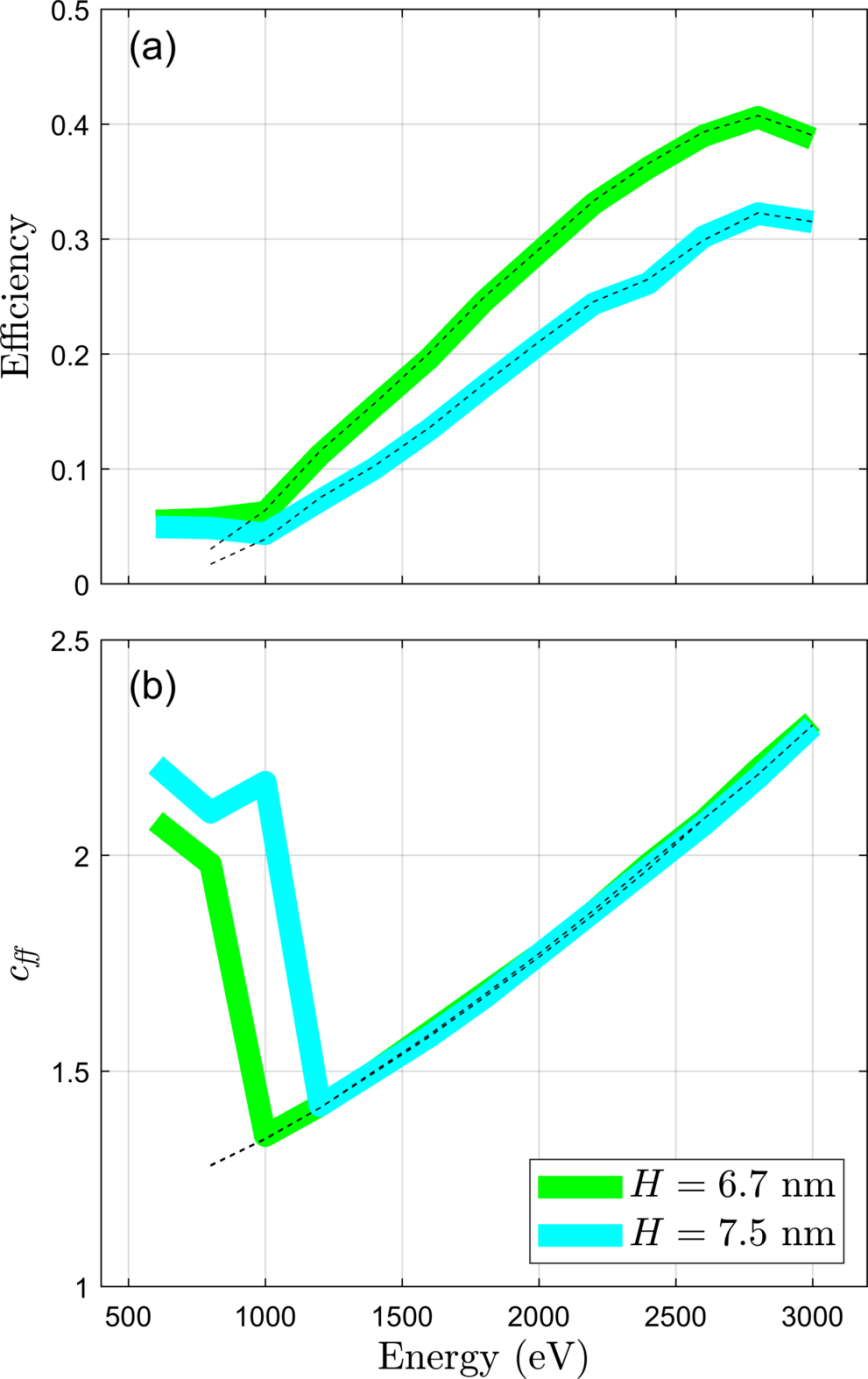
A summary of the performance of two different multilayer laminar gratings is presented. In (*a*) the peak grating efficiencies of the two gratings are plotted as a function of energy, while in (*b*) the corresponding values of *c*_ff_ are plotted versus energy. Solid lines are simulations performed in *MLgrating*, while the dashed lines were found using software developed at Tongji University based on *RETICOLO*. Both multilayer gratings are Cr/C multilayers on a silicon grating. The *H* = 7.5 nm grating is the same as that presented in Fig. 5 ▸, while the other grating is identical apart from *H* and Γ which are 6.7 nm and 0.67, respectively.

The peak multilayer laminar grating efficiencies from Fig. 5 ▸ are presented in Fig. 6 ▸(*a*), together with simulations performed for another multilayer laminar grating with very similar parameters. The corresponding dependence of *c*_ff_ on energy is also presented in Fig. 6 ▸(*b*). The dashed lines are corresponding simulations performed using a program developed at Tongji University, Shanghai, which at its core uses *RETICOLO* (Hugonin & Lalanne, 2005 ▸) to simulate the grating efficiencies. This program has been used to validate alternative analytical approaches for simulating multilayer gratings (Yang *et al.*, 2017 ▸; Huang *et al.*, 2020 ▸). The simulations using *MLgrating* and the alternative software are very similar above 1200 eV. Below this energy our simulations diverge because the simulations in *MLgrating* were performed over a wider range of *c*_ff_, and so here one observes at which energy the multilayer grating efficiency becomes larger than the ‘standard’ grating efficiency due to total external reflection.

**Figure 7:**
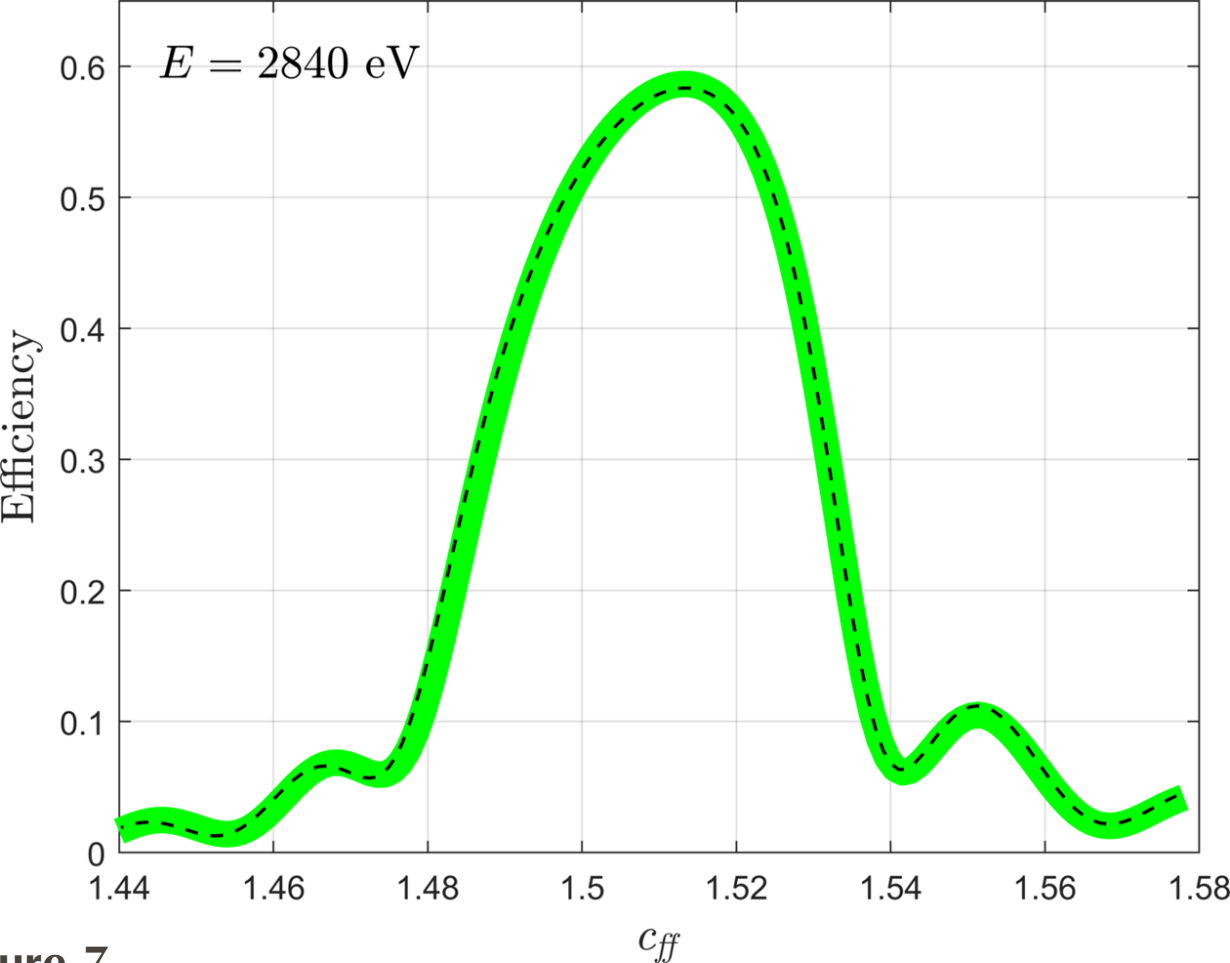
An example efficiency simulation of a multilayer blazed grating is presented as a function of *c*_ff_ at a single energy (*E* = 2840 eV). The solid line is a *MLgrating* simulation, while the dashed lines were found using software developed at Tongji University based on *RETICOLO*. The grating is a Cr/C multilayer on a silicon grating [see the corresponding schematic in Fig. 1 ▸(*b*)] with the following parameters: *N* = 1500 lines mm^−1^, *B* = 0.68°, *A* = 177.28°, *m* = −1, *d* = 5.87 nm, γ = 0.5 and *num*_*periods* = 40. The values of *m*_*max* and *num*_*slices* were 5 and 6, respectively.

In Fig. 7 ▸ we compare simulations for a multilayer blazed grating performed in *MLgrating* with those performed using the simulation software employed at Tongji University. We present simulations as a function of *c*_ff_ for an example multilayer blazed grating design. Here we chose to use a value of *m*_*max* = 5, although our convergence study showed little change in the simulated efficiencies even if *m*_*max* = 1 were used. Based on our earlier convergence study of the single-layer blazed grating (see Fig. 4 ▸ and corresponding text), we elected to use *num*_*slices* = 6. Once again, we find excellent agreement between *MLgrating* and a more established approach.

**Figure 8:**
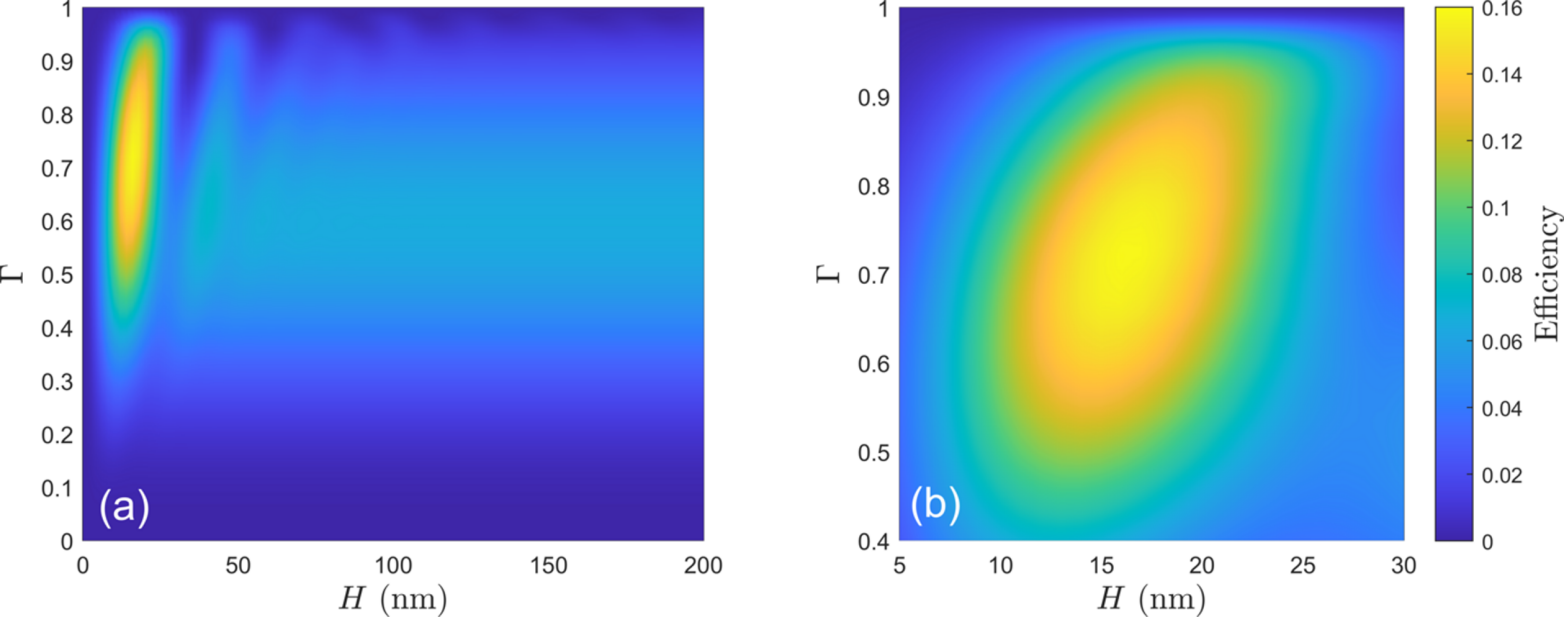
An example of how *MLgrating* could be used to optimize the design of a laminar multilayer grating. The grating was assumed to be made of rhodium, with *N* = 400 lines mm^−1^, *T* = 90°, *E* = 500 eV, *m* = −1 and *c*_ff_ = 2.25. *m*_*max* was set to 17. The parameters *H* and Γ were allowed to vary over a large range in panel (*a*), while the follow-up simulations presented in (*b*) were performed over a selected narrow range within which the grating efficiency is maximized.

To provide greater insight into how *MLgrating* could be used to optimize the design of a grating, we present a simplified example of its use in a script. In Fig. 8 ▸(*a*) we present the simulated grating efficiency of a laminar grating over the whole range of Γ (0 to 1) and over an extremely large range of *H*. Fig. 8 ▸(*b*) shows a follow-up set of simulations over selected smaller ranges in Γ and *H* to concentrate on the parameter space where the grating efficiency is maximized. It took less than 10 min to perform all the simulations presented in Fig. 8 ▸ on a typical modern laptop. The straightforward scripting of grating efficiency simulations facilitated in *MLgrating* enables the designer of the grating to use their time more effectively while also ensuring that the available parameter space is thoroughly explored.

## 3. Conclusions and outlook

We have systematically benchmarked *MLgrating* by comparing its output and performance with those of established grating simulation software. Our program provides a robust method for simulating both single-layer gratings as well as multilayer gratings for X-ray beamlines. As the code is trivially scriptable within MATLAB, it provides an easy-to-use tool for X-ray beamline designers to optimize their designs of (multilayer) gratings. We hope, by making the underlying MATLAB code freely available, that collaborators from around the world will be interested in helping to develop and extend the software, ensuring that the program continues to be competitive and also reflects the needs of X-ray beamline designers into the future.

In an open-source project such as *MLgrating*, there may be interest in creating a graphical user interface in MATLAB to make the program more user-friendly to users who are not familiar with MATLAB or other similar programming languages. Our preference so far has been to keep the code as simple as possible, with new users advised to modify the example simulation scripts provided for their own purposes.

An important future extension of *MLgrating* will be to enable the optional definition of underlayers and/or overlayers relative to the (multilayer) coating. This will provide an easy method to include binding layers, which are likely to be a different thickness to other coating layers. We would like to highlight that it is already possible in *MLgrating* to define a binding layer for a single-layer coating. While the impact of a binding layer on the efficiency of a multilayer grating will be very small in most practical applications, any overlayer is much more likely to have a significant effect on its performance. This extension of the program would also allow multilayer structures which have an ABAB…ABA structure to be simulated (at present *MLgrating* can only simulate ABAB…AB structures).

The current version of *MLgrating* cannot account for interfacial roughness, unlike both *REFLEC* and *PCGrate*. Within the framework imposed by *GD-Calc*, a graded-interface approach could be introduced. This would work in a similar way to the corresponding approach used in the multilayer simulation program *IMD* (Windt, 1998 ▸). However, this would almost certainly require the value of *num*_*slices* to be increased, impacting on the speed of the program.

Another future possibility for *MLgrating* would be to compile the code using *MATLAB Compiler* so that an executable can be created that can be run without a MATLAB installation, or alternatively use *MATLAB Compiler SDK* to integrate the code with programs written in other languages. The latter option is of particular interest if one would like to combine *MLgrating* with ray-tracing software such as *SHADOW* (Sanchez del Rio *et al.*, 2011 ▸). This would be an important step in enabling the photon flux of a PGM-based beamline to be accurately simulated without needing to combine results from multiple simulation programs independently.

## Figures and Tables

**Table 1:** Input parameters for *MLgrating*

	Parameter name	Required for laminar, blazed or both grating types
List of grating materials	*materials*	Both
Groove density (grooves mm^−1^)	*N* (*N* = 1/*D*)	Both
Multilayer period (nm)	*d*	Both
First layer thickness/multilayer period	γ	Both
Number of periods in multilayer	*num*_*periods*	Both
Number of slices in groove profile	*num*_*slices*	Both
Maximum diffraction order	*m*_*max*	Both
Groove height (nm)	*H*	Laminar
Groove width/groove period *D*	Γ	Laminar
Trapezoidal angle (°)	*T*	Laminar
Blaze angle (°)	*B*	Blazed
Apex angle (°)	*A*	Blazed
Photon energy (eV)	*E*	Both
Grating diffraction order	*m*	Both
Grating *c*_ff_ = 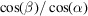	*c*_ff_	Both

**Table 2:** Speed testing of *MLgrating* compared with *REFLEC* The grating simulated is the same as presented in Fig. 4 ▸. In each case, simulations were performed for 100 photon energies.

Software	*num*_*slices*	Time (s)
*REFLEC*	N/A	∼28
*MLgrating*	1	5.0
*MLgrating*	4	8.0
*MLgrating*	10	15.0
*MLgrating*	20	27.4
*MLgrating*	50	62.4
